# Using Virtual Reality to Improve Antiretroviral Therapy Adherence in the Treatment of HIV: Open-Label Repeated Measure Study

**DOI:** 10.2196/13698

**Published:** 2019-06-20

**Authors:** Omer Liran, Robert Dasher, Kevin Kaeochinda

**Affiliations:** 1 Department of Psychiatry and Biobehavioral Sciences David Geffen School of Medicine at University of California - Los Angeles Los Angeles, CA United States; 2 Department of Psychology Marymount California University Rancho Palos Verdes, CA United States

**Keywords:** HAART, technology, virtual reality, medication adherence, viral load, education

## Abstract

**Background:**

Nonadherence to HIV medications is a serious unsolved problem and is a major cause of morbidity and mortality in the HIV-positive population. Although treatment efficacy is high if compliance is greater than 90%, about 40% of people with HIV do not meet this threshold.

**Objective:**

This study aimed to test a novel approach to improve medication adherence by using a low-cost virtual reality (VR) experience to educate people with HIV about their illness. We hypothesized that people with HIV would be more likely to be compliant with the treatment following the 7-minute experience and, therefore, should have decreasing viral load (VL), increasing cluster of differentiation 4^+^ (CD4^+^) cell counts, and improved self-reported adherence.

**Methods:**

We showed the VR experience to 107 participants with HIV at a county hospital in Los Angeles, California. Participants were asked to self-report how often they take their medications on a Likert-scale. The self-reported question (SRQ) was given before and at least 2 weeks after the VR experience. We also compared VL and CD4^+^ cell counts before and on average 101 days after the experience. VL and CD4^+^ were obtained per the clinic’s standard care protocol. Two-tailed paired *t* tests were performed on the initial and follow-up SRQ scores, VL, and CD4^+^. We restricted the CD4^+^ analysis to participants who had a pre-CD4^+^ below normal (defined as 500 cells/mm^3^). To reduce the possibility that VL were trending down and CD4^+^ were trending up regardless of the VR experience, 2 serial VL and CD4^+^ obtained before the experience were also compared and analyzed. Immediately following the VR experience, participants were given a 4-question Likert-type postexperience questionnaire (PEQ) that assessed their opinions about the experience.

**Results:**

SRQ scores improved from pre to post experience with high significance (*P*<.001). VL decreased from pre to post experience by 0.38 log_10_ copies/mL (95% CI 0.06-0.70; *P*=.02). In contrast, the 2 serial VL obtained before the experience showed no statistically significant changes. There was also a statistically significant increase in CD4^+^ (95% CI –3.4 to –54.3 cells/mm^3^; *P*=.03). Analysis of the PEQ revealed that VR was comfortable for almost all of the participants and that most participants believed the experience to be educational and that it would improve their medication adherence.

**Conclusions:**

The findings suggest that the low-cost VR experience caused an increased rate of antiretroviral therapy adherence that resulted in a decrease of VL and an increase of CD4^+^. Further studies are required to explore the duration of this effect and whether these results are generalizable to other treatment settings and populations.

## Introduction

### Background

HIV is a serious medical illness with significant morbidity and mortality. Advances in treatment have continued to progress with improved regimens both in terms of efficacy and tolerability and in terms of convenience in the form of combination medications in a once- or twice-daily pill known as highly active antiretroviral therapy (HAART). Adherence to HAART has been shown to be correlated with survival [1,2] and quality of life [3]. Primarily because of HAART, HIV is now widely regarded as a chronic and manageable infection [4]. Treatment efficacy, defined as a decrease in viral load (VL), is highly correlated with an almost perfect adherence rate to HAART of at least 90 to 95% [5,6]. However, about 40% of HIV-positive patients have adherence rates to HAART below 90% [7,8].

Several factors have been identified that correlate to poor medication adherence including homelessness [9-11], comorbid mental illness [12,13], and active substance use [14,15]. In addition, health literacy has been shown to be an independent predictor for HAART adherence [16,17]. A survey of Medicare patients revealed that 34% of English-speaking and 54% of Spanish-speaking respondents had inadequate or marginal health literacy [18]. Compounding to this challenge, a study found that although physicians frequently believe that they are using nontechnical terms when communicating with their patients, they actually used nontechnical language in only 12% of encounters [19]. Moreover, a study of audiotaped encounters between patients and physicians revealed that there was a discussion about the patient’s degree of understanding in only 2% of the encounters [20]. These data suggest that changes to how patients are educated about their illness may be needed to optimize medication adherence rates.

Virtual reality (VR) has recently emerged as an effective tool in several branches of medicine [21-26]. Recent advances in VR head-mounted displays (HMDs) have made their use increasingly comfortable, affordable, and immersive [27]. VR radically differs from most other media because it can create a level of immersion that produces a feeling of presence—a “sense of being there” [28]. Due to this distinct experiential quality, VR can augment learning with an immersive experience. In addition, VR has been shown to improve learning outcome gains [29-31], and, compared with educational videos, VR has been shown to increase engagement, positive emotions, and remembering of the presented information [32,33]. In fact, an increasing number of classrooms are utilizing VR technology to provide unique and effective educational experiences for their students [34,35].

### Objectives

In light of this trend, the aim of this study was to investigate the effects of an educational VR experience on HAART adherence in people with HIV. Specifically, people with HIV were enrolled at a county clinic to undergo an interactive VR experience that educates them about the importance of HAART adherence. The participants were asked to self-report how often they take their HAART medications on a Likert-scale. To verify the self-reported compliance, pre- and post- HIV VL and CD4^+^ cell counts were obtained from the participants’ digital chart. In addition, second pre- VL and CD4+ were obtained to detect any trends before the VR experience. Participants also completed a post experience questionnaire (PEQ; Multimedia Appendix 1) that assessed their immediate reaction to the VR experience with regard to its novelty, comfort, educational value, and perceived effectiveness.

## Methods

### Participants

From February to September 2018, 107 HIV-infected participants were recruited from Olive View-University of California, Los Angeles (UCLA) Medical Center’s HIV clinic in Los Angeles, California. To enroll, participants had to be diagnosed with HIV, be on HAART, be at least 18 years of age, be fluent in English or Spanish, and have no major uncorrectable problems with vision or hearing. There were no selection criteria for initial VL, CD4^+^, or HAART regimen. Participants were continuously recruited from the clinic until the study’s predetermined time for access to the clinic elapsed. The institutional review board at Olive View-UCLA Medical Center approved the study. Each participant signed a written informed consent. There were no financial incentives for enrolling in the study.

### Interventions

A 7-minute interactive 3-dimensional educational VR experience was created that illustrates a simplified version of the mechanisms of immune cells, HIV, and antiretroviral medications. We used a Dell Windows Mixed Reality HMD. Research assistants helped place the HMD on the participants’ head and adjusted it for optimal clarity and comfort. Participants required no training before the VR experience. The English and Spanish versions of the VR experience had different narrators but otherwise all participants had the same VR experience.

The narrator begins by introducing a virtual person with HIV named Dave and explaining that Dave is made up of cells. A magnifying glass moves to show Dave’s skin cells (Multimedia Appendix 2). The experience then virtually transports the participant into one of Dave’s arteries. Red blood cells can be seen traveling through the artery when a single rod-shaped bacterium swims nearby (Multimedia Appendix 3). For dramatic visual effect, the bacterium is seen releasing green particles representing poison into the artery. Intense music ensues when a white blood cell (WBC) chases the bacterium, which it eventually engulfs (Multimedia Appendix 4). A single HIV then enters the scene, gets inserted into the WBC, and makes many copies of itself until the WBC bursts (Multimedia Appendix 5). With no WBCs around, many bacteria swim freely and release the green poisonous particles. The artery gradually turns green, signifying that Dave is sick (Multimedia Appendix 6). The scene resets and the narrator says, “Let’s see what happens when Dave remembers to take his medications.” Hopeful music plays and the narrator encourages the participant to press a button to release “a dose of life-saving medication.” When the participant presses the button, medication in the form of gold particles enters the artery and creates a shield around the WBC (Multimedia Appendix 7). HIV again tries to attack the WBC but gets repulsed by the shield. The participant is asked to press the button several more times to give more medication as more HIV attempt to attack the WBC (Multimedia Appendix 8). In conclusion, the narrator explains that although Dave feels fine, he is required to continue taking his medications to keep the shield active.

### Data Collection

Participants were asked to self-report how often they take their medications on a Likert-scale. This SRQ was asked just before the VR experience and again at least 2 weeks post experience. The initial SRQ was asked in a face-to-face interview, while the follow-up was asked either by phone or with another face-to-face interview. Immediately following the experience, a PEQ was administered.

VL and CD4^+^ cell counts were drawn as per standard clinic protocol. Both the treating physician who ordered the labs and the laboratory were blinded as to which patients were enrolled in the study. Post-, pre-, and second pre-VL/CD4^+^ were recorded from the participants’ digital chart. Given that blood concentrations of HIV ribonucleic acid are expected to decrease rapidly in the first 2 weeks after HAART initiation [36,37], post-VL/CD4^+^ were defined as the first labs that were obtained at least 14 days post experience, whereas pre-VL/CD4^+^ were defined as the most recent labs that were obtained before or on the same day as the experience. Finally, second pre-VL/CD4^+^ were defined as the most recent labs that were obtained before the pre-VL/CD4^+^. All identifying information was removed to protect the privacy of the participants. Demographic data obtained from each participant were age, gender, and primary spoken language.

### Data Analysis

All analyses were performed by use of IBM SPSS software, version 22. The raw VL were converted to a logarithmic scale because of the large variance in the data. Spearman correlation was performed on the initial SRQ scores and pre-VL. A 2-tailed paired *t* test analysis was performed to compare the initial and follow-up SRQ scores. A 2-tailed paired *t* test was also performed to compare pre- to post-VL, and pre- to post-CD4^+^. To assess for the possibility that the VL were decreasing even before the VR experience, a 2-tailed paired *t* test was performed on the second pre-VL and pre-VL. For the CD4^+^ analysis, only participants who had below normal pre-CD4^+^ (defined as 500 cells/mm^3^) were included. Participants who did not have pre-VL, second pre-VL, or post-VL were excluded from this analysis. Finally, the PEQ was correlated with age using Spearman correlation.

## Results

Descriptive statistics were computed and compiled in Table 1. There were no significant gender- or age-related differences in terms of SRQ scores or VL. Out of 107 participants, 28 were excluded because 11 did not have a second pre-VL and 17 did not have a post-VL recorded by the time data collection has concluded. There were no demographic differences between the 28 excluded participants and the remaining 79 participants.

Out of the 79 participants, 4 did not complete the initial SRQ correctly and therefore were excluded from SRQ statistics. Of the remaining 75 participants, 69% (52/75) were identified as high adherers (score 5/5 on Likert-scale), 15% (11/75) as medium adherers (score 4), and 16% (12/75) as low adherers (score <4). A total of 8 participants did not complete the follow-up SRQ because they were both unreachable by phone and were unavailable for follow-up face-to-face interviews. Out of 67 participants who completed the follow-up SRQ, 90% (60/67) were identified as high adherers, 6% (4/67) as medium adherers, and 4% (3/67) as low adherers. The initial SRQ scores were shown to be correlated with pre-VL (n=75, *r*_s_=0.316; *P*=.006). Likewise, the follow-up SRQ scores were shown to be correlated with post-VL (n=65, *r*_s_=0.549; *P<*.001). SRQ scores improved very significantly (95% CI .24-.70, *P<*.001) from pre to post VR experience. Table 2 summarizes participants’ transitions from pre to post SRQ. There were not enough data to analyze statistical differences between the 2 modalities used to collect the SRQ (face-to-face and phone interviews); however, other studies suggest that they should be highly correlated [38-40].

**Figure 1:**
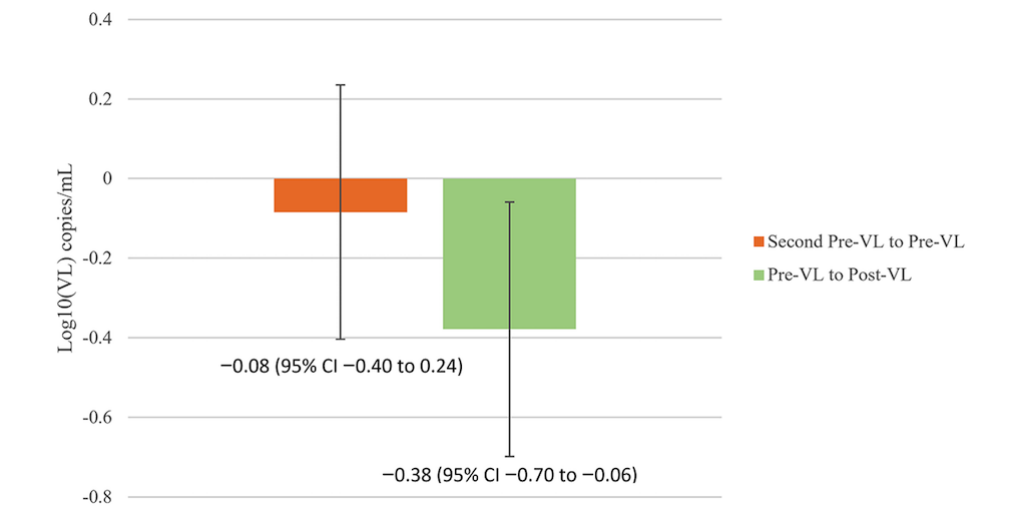
The change log10(VL) from before to after the virtual reality experience was significant (green, P=.02). On the other hand, the change from the 2 viral loads prior to the experience was nonsignificant (orange, P=.62). The difference between the 2 measurements is highly significant (P=.01). VL: viral loads.

Comparison of pre-VL to post-VL showed an average decrease of 0.38 log_10_ copies/mL (95% CI 0.06-0.70; *P*=.02). On the other hand, analysis of second pre-VL and pre-VL showed no statistically significant changes (95% CI –0.40 to 0.24; *P*=.62; Figure 1). The difference between these 2 measurements was highly significant (*P*=.01). The medians for second pre-, pre-, and post-VL were all 0.

When taken as a whole, there was no statistically significant difference in pre- to post-CD4^+^. However, when only participants who had a pre-CD4^+^ below normal (defined as 500 cells/mm^3^) were included in the analysis, there was a statistically significant increase from pre- to post-CD4^+^ (95% CI –54.3 to –3.4; *P*=.03). Conversely, there was no significant CD4^+^ count difference between second-pre and pre-CD4^+^ (95% CI –56.2 to 42.6; *P*=.78). The medians for second pre-, pre-, and post-CD4^+^ were 285, 301, and 323, respectively.

On average, the pre-VL were drawn 60 days (SD=74) before the VR experience. A total of 10 participants had their pre-VL drawn on the day of the VR experience, thereby accounting for this non-normal distribution. The post-VL were drawn on average 101 days (SD=62) after the VR experience. The average interval from second pre-VL to pre-VL was 141 days (SD=90).

None of the questions on the PEQ were significantly correlated with age. None of the participants reported that the VR experience was uncomfortable. Only 2 participants disagreed that they learned something new about their immune system, HIV, or their medications. Finally, 100 participants (94%) agreed or strongly agreed that they are more likely to take their medications because of the VR experience (Table 3).

**Table 1:** Demographic characteristics of the overall sample and the analyzed group. There were no statistically significant group differences in gender, age, or language.

Demographics		Overall sample (N=107), n (%)	Analyzed group (n=79), n (%)
**Gender**			
	Male	83 (77.6)	59 (75)
	Female	24 (22.4)	20 (25)
**Age (years)**			
	18-39	31 (29.0)	25 (32)
	40-49	29 (27.1)	21 (27)
	50-59	34 (31.8)	26 (33)
	60+	13 (12.1)	7 (9)
**Language**			
	English	100 (93.5)	74 (94)
	Spanish	7 (6.5)	5 (6)

**Table 2:** Number of patients transitioning from low, medium, or high adherence groups in the pre self-reported question (SRQ) to the post SRQ (n=67). High adherence was defined as SRQ score 5/5; medium adherence was defined as SRQ score 4; low adherence was defined as SRQ score <4.

Self-Reported Questionnaire adherence score	Post low, n (%)	Post medium, n (%)	Post high, n (%)	Total, n (%)
Pre low	3 (4)	1 (1)	8 (12)	12 (18)
Pre medium	0 (0)	3 (4)	6 (9)	9 (13)
Pre high	0 (0)	0 (0)	46 (69)	46 (69)
Total	3 (4)	4 (6)	60 (90)	67 (100)

**Table 3:** Responses to the Postexperience Questionnaire (PEQ). Question 1: Virtual Reality is a new experience for me; Question 2: The experience was comfortable; Question 3: I learned something new about my immune system; Question 4: I am now more likely to take my HIV medications. N=106.

Post-Experience Questionnaire	Question 1, n (%)	Question 2, n (%)	Question 3, n (%)	Question 4, n (%)
Agree or strongly agree	91 (86)	105 (99)	92 (87)	100 (94)
Strongly disagree, disagree, or neutral	15 (14)	1 (1)	14 (13)	6 (6)

## Discussion

### Principal Findings

When taken together, several points of evidence strongly suggest that participants indeed were more compliant with their HAART medications because of the VR experience: (1) the SRQ scores were correlated with VL, (2) the SRQ scores improved from pre to post experience, (3) the VL decreased from pre to post experience but not from serial VL obtained before the experience, (4) the CD4^+^ cell counts improved from pre to post experience but not from serial counts obtains before the experience, and (5) almost all participants (94%) answered on the PEQ that they are more likely to take their medications because of the VR experience.

### Limitations

Although these preliminary results are promising, a number of limitations need to be addressed. First, the study did not randomize participants and no controls were used. Future studies should ideally use a placebo-controlled design. Second, the study was conducted only at a county clinic and therefore may not necessarily be generalizable to other settings or populations. Third, the VL and CD4^+^ counts were obtained as per the clinic’s standard of care. A superior study would control the timing of the blood draws to more thoroughly account for extraneous variables in the data. Fourth, the duration for which increased HAART adherence is maintained is unclear. A longitudinal study with more participants would allow for more robust statistical analyses. It would also be worth testing whether repeating the experience every few months can maintain improved compliance over time. Finally, collecting further demographic data on, for example, health literacy, homelessness, substance use, and comorbid mental illness could yield interesting correlations that may help advise which subpopulations would be most likely to benefit from the VR experience.

### Conclusions

This study suggests that patient education using VR is effective for increasing HAART adherence. If the results are confirmed, VR’s effectiveness and relative low cost offers a great opportunity for clinics to implement a simple solution that may improve both the morbidity and mortality of their patients. Future studies should attempt to generalize the results to other settings, populations, and illnesses—especially those in which patients feel healthy and may not feel the need for taking their medications as scheduled. Compared with other traditional media platforms such as videos, VR is more immersive and thus has a greater positive emotional impact, which can improve engagement and learning. VR is rapidly becoming more accessible, affordable, and immersive, and more studies are needed to further explore opportunities for using this maturing technology for improving peoples’ lives.

## Appendix

Multimedia Appendix 1 Postexperience questionnaire.

Multimedia Appendix 2 Dave’s skin is made up of cells.

Multimedia Appendix 3 A bacterium swims inside an artery.

Multimedia Appendix 4 A white blood cell engulfs the poison-producing bacterium.

Multimedia Appendix 5 HIV enters the white blood cell, makes copies of itself, and causes the cell to explode.

Multimedia Appendix 6 Without the white blood cells, bacteria can produce poison without deterrence.

Multimedia Appendix 7 The medication produces a shield around the white blood cell.

Multimedia Appendix 8 HIV cannot penetrate the medicated white blood cell.

## Abbreviations

CD4+: cluster of differentiation 4+
HAART: highly active antiretroviral therapy
HMD: head-mounted display
PEQ: post experience questionnaire
SRQ: Self-reported question
UCLA: University of California, Los Angeles
VL: viral load
VR: virtual reality
WBC: white blood cell
