# Supplementary material for: Using Virtual Reality to Improve Antiretroviral Therapy Adherence in the Treatment of HIV: Open-Label Repeated Measure Study
Source: Interact J Med Res. 2019 Jun 20;8(2):e13698. doi: 10.2196/13698 (PMC6610452; doi:10.2196/13698)
Supplement: Multimedia Appendix 1 [file ijmr_v8i2e13698_app1.pdf]

Postexperience Questionnaire

ID Number: ___________________________

Date: _________________________________

	Strongly Disagree	Disagree	Neutral	Agree	Strongly Agree
Virtual Reality is a new experience for me.					
The experience was comfortable.					
I learned something new about my immune system, HIV, and/or my medications.					
I am now more likely to take my HIV medications.					
