# Supplementary figures and images for: Using Virtual Reality to Improve Antiretroviral Therapy Adherence in the Treatment of HIV: Open-Label Repeated Measure Study
Source: Interact J Med Res. 2019 Jun 20;8(2):e13698. doi: 10.2196/13698 (PMC6610452; doi:10.2196/13698)

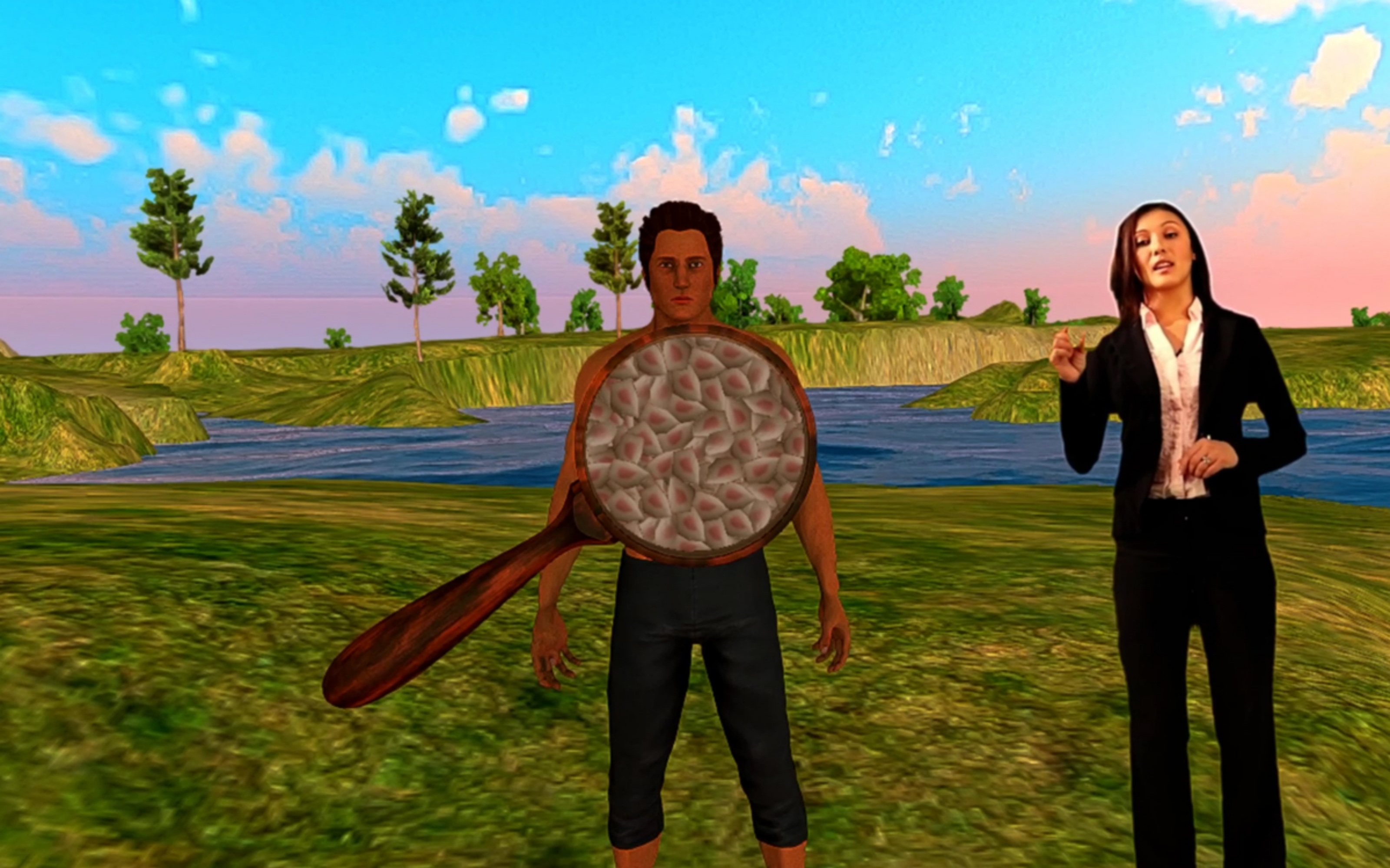

Supplement: Multimedia Appendix 2 [file ijmr_v8i2e13698_app2.pdf]

**BACTERIA**

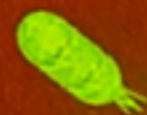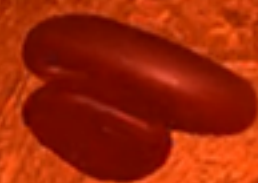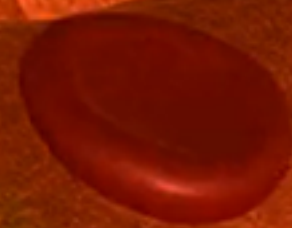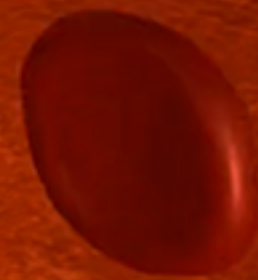

Supplement: Multimedia Appendix 3 [file ijmr_v8i2e13698_app3.pdf]

**IMMUNE CELL**

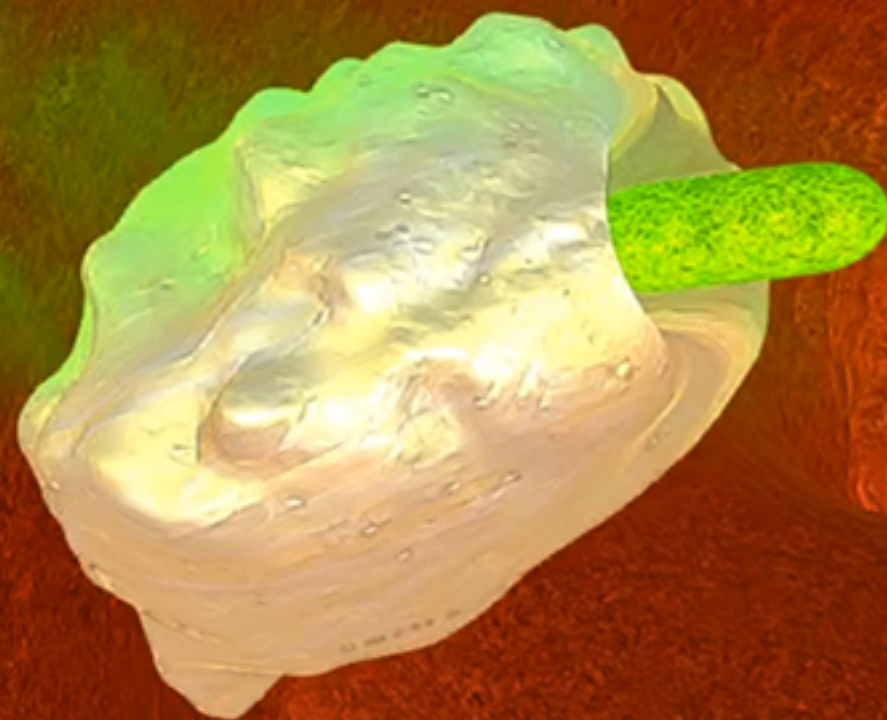

Supplement: Multimedia Appendix 4 [file ijmr_v8i2e13698_app4.pdf]

HIV

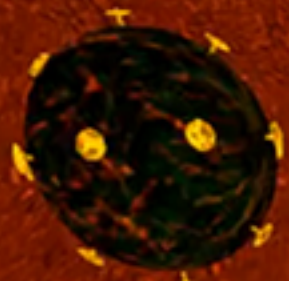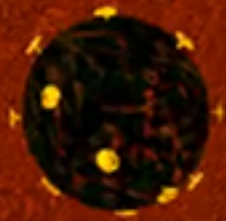

HIV

HIV

HIV

HIV

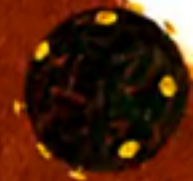

HIV

HIV

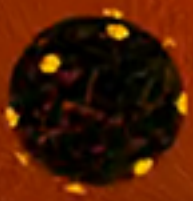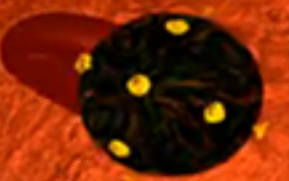

HIV

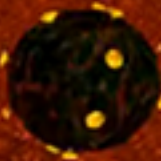

HIV

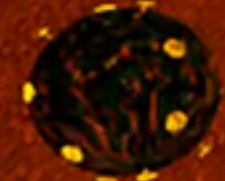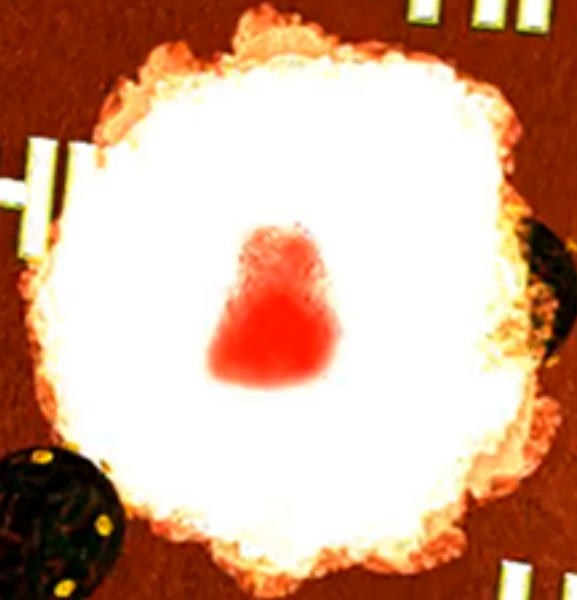

Supplement: Multimedia Appendix 5 [file ijmr_v8i2e13698_app5.pdf]

BACTERIA  
HIV

HIV

HIV

HIV

HIV

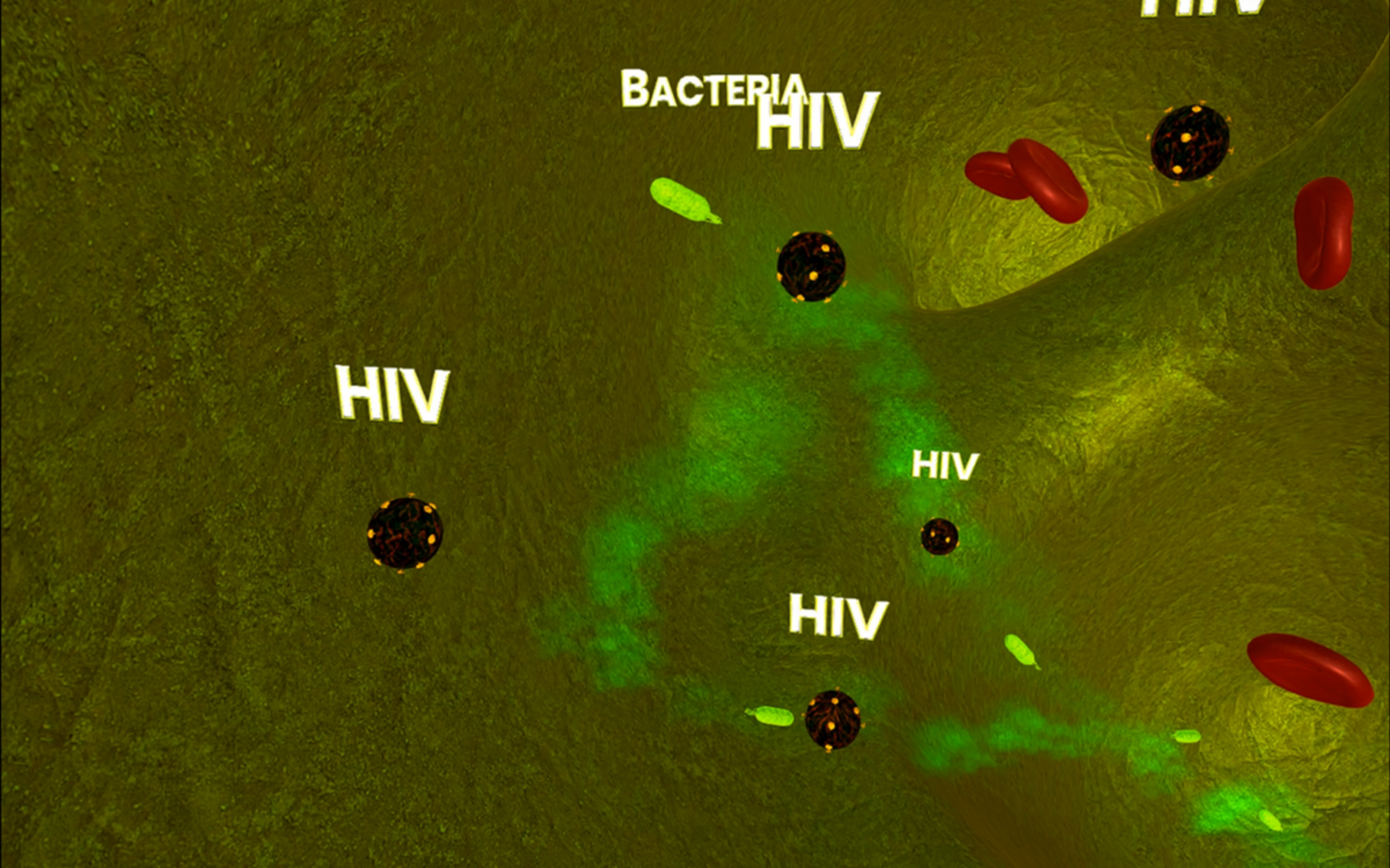

Supplement: Multimedia Appendix 6 [file ijmr_v8i2e13698_app6.pdf]

MEDICATED IMMUNE CELL

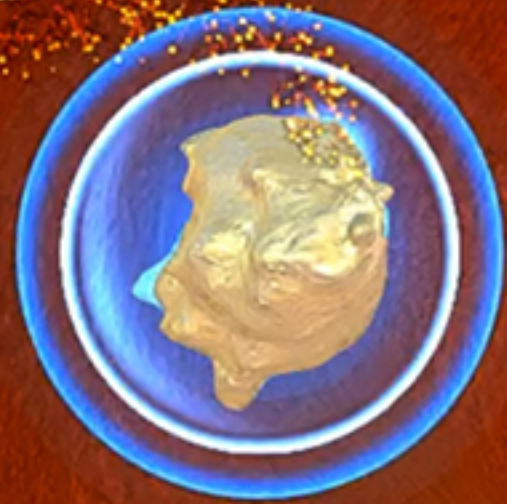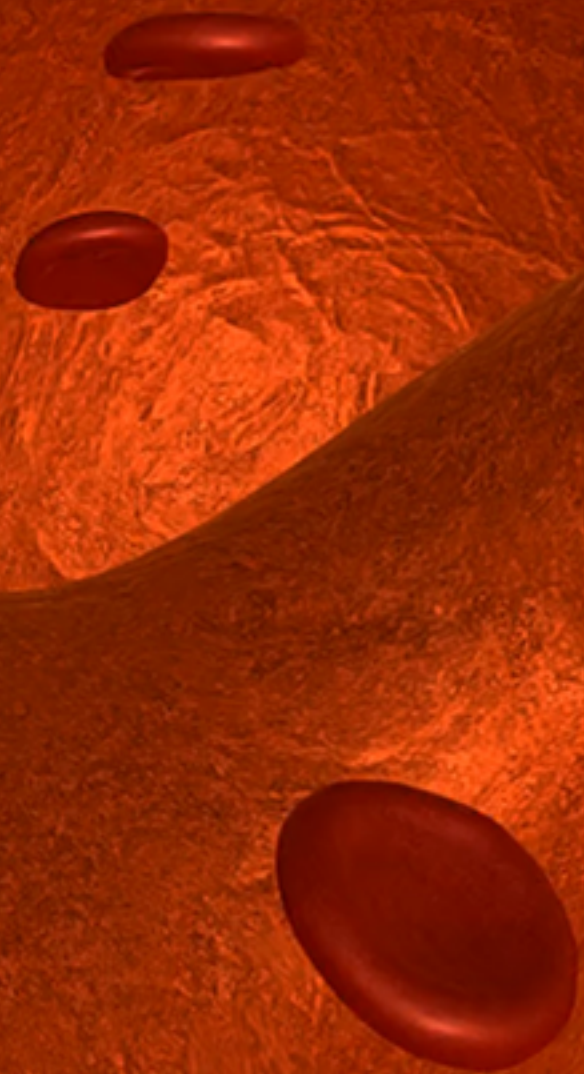

Supplement: Multimedia Appendix 7 [file ijmr_v8i2e13698_app7.pdf]

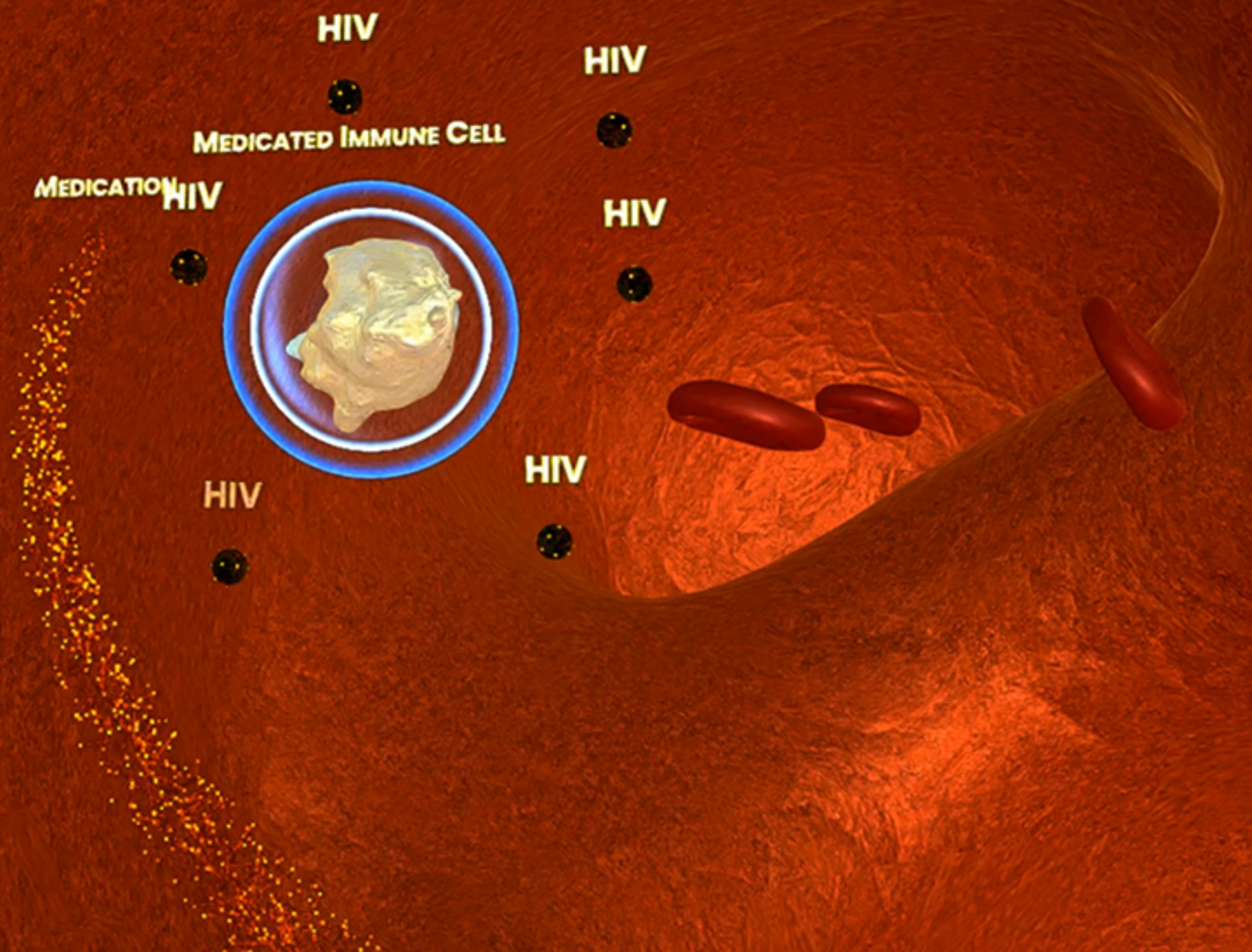

Supplement: Multimedia Appendix 8 [file ijmr_v8i2e13698_app8.pdf]
